# Exploring the potential of artificial intelligence in improving skin lesion diagnosis in primary care

**DOI:** 10.1038/s41598-023-31340-1

**Published:** 2023-03-15

**Authors:** Anna Escalé-Besa, Oriol Yélamos, Josep Vidal-Alaball, Aïna Fuster-Casanovas, Queralt Miró Catalina, Alexander Börve, Ricardo Ander-Egg Aguilar, Xavier Fustà-Novell, Xavier Cubiró, Mireia Esquius Rafat, Cristina López-Sanchez, Francesc X. Marin-Gomez

**Affiliations:** 1 Centre d’Atenció Primària Navàs-Balsareny, Institut Català de la Salut, Navàs, Spain; 2 Health Promotion in Rural Areas Research Group, Gerència Territorial de la Catalunya Central, Institut Català de la Salut, Sant Fruitós de Bages, Spain; 3 Dermatology Department, Hospital de la Santa Creu i Sant Pau, Universitat Autònoma de Barcelona, Barcelona, Spain; 4 Dermatology Associate Research Group, Institut d’Investigació Biomèdica Sant Pau (IIB SANT PAU), Barcelona, Spain; 5 Unitat de Suport a la Recerca de la Catalunya Central, Fundació Institut Universitari per a la Recerca a l’Atenció Primària de Salut Jordi Gol i Gurina, Sant Fruitós de Bages, Spain; 6 Factulty of Medicine, University of Vic-Central University of Catalonia, Vic, Spain; 7 iDoc24 Inc, San Francisco, CA USA; 8 Institute of Clinical Sciences, University of Gothenburg, Sahlgrenska, Gothenburg, Sweden; 9 Fundació Althaia de Manresa, Manresa, Spain; 10 Servei de Dermatologia, Hospital Universitari Mollet, Mollet del Vallès, Barcelona, Spain; 11 Servei d’Atenció Primària Osona, Gerència Territorial de la Catalunya Central, Institut Català de La Salut, Vic, Spain

**Keywords:** Skin diseases, Health care

## Abstract

Dermatological conditions are a relevant health problem. Machine learning (ML) models are increasingly being applied to dermatology as a diagnostic decision support tool using image analysis, especially for skin cancer detection and disease classification. The objective of this study was to perform a prospective validation of an image analysis ML model, which is capable of screening 44 skin diseases, comparing its diagnostic accuracy with that of General Practitioners (GPs) and teledermatology (TD) dermatologists in a real-life setting. Prospective, diagnostic accuracy study including 100 consecutive patients with a skin problem who visited a participating GP in central Catalonia, Spain, between June 2021 and October 2021. The skin issue was first assessed by the GPs. Then an anonymised skin disease picture was taken and uploaded to the ML application, which returned a list with the Top-5 possible diagnosis in order of probability. The same image was then sent to a dermatologist via TD for diagnosis, as per clinical practice. The GPs Top-3, ML model’s Top-5 and dermatologist’s Top-3 assessments were compared to calculate the accuracy, sensitivity, specificity and diagnostic accuracy of the ML models. The overall Top-1 accuracy of the ML model (39%) was lower than that of GPs (64%) and dermatologists (72%). When the analysis was limited to the diagnoses on which the algorithm had been explicitly trained (n = 82), the balanced Top-1 accuracy of the ML model increased (48%) and in the Top-3 (75%) was comparable to the GPs Top-3 accuracy (76%). The Top-5 accuracy of the ML model (89%) was comparable to the dermatologist Top-3 accuracy (90%). For the different diseases, the sensitivity of the model (Top-3 87% and Top-5 96%) is higher than that of the clinicians (Top-3 GPs 76% and Top-3 dermatologists 84%) only in the benign tumour pathology group, being on the other hand the most prevalent category (n = 53). About the satisfaction of professionals, 92% of the GPs considered it as a useful diagnostic support tool (DST) for the differential diagnosis and in 60% of the cases as an aid in the final diagnosis of the skin lesion. The overall diagnostic accuracy of the model in this study, under real-life conditions, is lower than that of both GPs and dermatologists. This result aligns with the findings of few existing prospective studies conducted under real-life conditions. The outcomes emphasize the significance of involving clinicians in the training of the model and the capability of ML models to assist GPs, particularly in differential diagnosis. Nevertheless, external testing in real-life conditions is crucial for data validation and regulation of these AI diagnostic models before they can be used in primary care.

## Introduction

Skin diseases are one of the main reasons for consultation in Primary Care (PC)^1^. To give an example, in the United States, each person has on average, 1.6 skin diseases per year^1–3^. Approximately 7.6% of the population of Catalonia consults PC annually for skin lesions^4^, generating 35% of referrals to dermatology^5^. However, the diagnostic accuracy of general practitioners in dermatological diseases is highly variable, around 48–77%^6,7^.

TD involves storing and transmitting photographs of skin lesions and text through the Internet. The use of TD as a consultation tool for dermatology services in PC is now common. It is estimated that more than 70% of all people with a skin problem in PC can be seen by TD and do not need to be referred to an in-person dermatologist^8,9^. This is a good sorting method, particularly for skin cancer^10,11^. TD has been shown to avoid unnecessary travel, decrease waiting time, provide diagnostic support at the time of the visit, and increase both user and provider satisfaction^9,12–16^.

The 4th industrial revolution^17^ and the application of artificial intelligence (AI) in the healthcare field open a door to more efficient, individualised and preventive medicine. There are currently several fields of medicine in which these new technologies help in the management of various diseases, such as screening for diabetic retinopathy, reading radiological images, or assisting during endoscopies, among others^18,19^.

Medical images are the most widely used data format in AI development^20^. In recent years there has been a substantial improvement in this field, especially applied to the automatic classification of medical images, through deep learning techniques using convolutional neural networks (CNN). In some cases, the performances are comparable to those achieved by medical specialists. In dermatology, ML using image recognition is especially developed in skin cancer screening^21–24^. More recently, its use has been extended to a wider range of skin lesions, such as inflammatory and infectious lesions^25–28^, and also in the recognition of cutaneous manifestations of COVID-19^29^. This suggests that its use in PC as a diagnostic support and screening tool for consultations related to skin problems would standardise and improve the effectiveness and efficiency of the professionals working there.

Some of these tools generate a list of differential diagnoses that can help the GP to broaden their range of diagnoses and therapeutic approaches to the assessed lesion. The fact that the algorithm can give 5 diagnoses from a single image means that the clinician can not only arrive at the final diagnosis, but can also consider alternative diagnoses that may condition the follow-up to ensure that the lesion is developing correctly.

For example, an inflammatory lesion may lead to a diagnosis of dermatitis, ringworm, pityriasis, psoriasis, neurodermatitis. These entities are different in themselves but for some of which the therapeutic approach is similar. Another example is a warty lesion, which can make the differential diagnosis between a viral wart, but also between other entities such as seborrheic keratosis and pathologies with malignant potential such as actinic keratosis and also carcinomas. However, although diagnostic yields are very high in silico, there have been few studies performed in routine clinical practice settings employing non-standardised imaging, so validation of these tools prospectively in real life is imperative. In Europe, the current governing regulation is the Medical Device Regulation (Regulation 2017/745)^30^, which has been in vigour since May 2020 and repeals Directive 93/42^31^. This new regulation introduced new responsibilities for the European Medicines Agency (EMA) and national authorities competent in the evaluation of certain categories of medical devices. The new regulation stipulates that manufacturers ensure that devices meet a number of essential requirements that depend on the potential risk of each device and require accreditation by an independent body. Thus, in the case of the application of ML model as a complementary diagnostic tool, different groups of experts around the world have developed guidelines to stipulate the essential requirements to be assessed in this practice. Several studies agree that prospective studies, such as the present study, are necessary to confirm that the application of these algorithms in clinical practice works, and to evaluate their potential impact^32–36^.

Although it is in PC where most consultations related to skin conditions are first received, there have been few studies performed in this setting. Some studies have included PC GPs along with dermatologists as image readers to compare the performance of the models with that of the professionals^37^. Other studies have concluded that AI tools could be used in PC, resulting in a new tool for diagnostic support, screening, and to extend differential diagnosis by non-expert professionals^37,38^. However, this has not been widely studied and the proof is insufficient.

Autoderm is a Class I CE marked DST in dermatology which uses ML to help diagnose skin lesions in a faster and more accurate way^39^. The current model can examine 44 different types of skin diseases, including inflammatory diseases, tumours, and genital skin problems, among others, representing 90% of the consultations made by the general population^1,3,4^. The model can be accessed through an Application Programming Interface (API) that can be integrated into any platform that is connected to the Internet. After examining a photograph, the model generates a ranking of the five skin diseases that have the highest concordance with the lesion shown in the photo, sorted in order of probability. Autoderm uses a set of 3 neural networks: resnet-18, resnet-34^40^ and squeezenet^41^, provided by TorchVision (PyTorch)^42^, which is used for applications such as computer vision and natural language processing. It was trained with an in-housedataset of 55,364 images in the training set and 13,841 for the test set. As for dermoscopic images, it was only trained with approximately 2000 images obtained from the HüD dermatoscope and other Dermlite dermatoscope models. These images were all taken by the layman or a healthcare worker using a smartphone. Data augmentation methods were used during algorithm training. This consists of modifying images in the training set (orientation, brightness, etc.) so that relevant information is not lost, but allowing the algorithm to be exposed to a more general distribution of data. After the data augmentation process, the number of images increased to approximately 120,000. The theoretical diagnostic accuracy of the model tested is 49.3% (Top-1), 70.1% (Top-3) and 81.7% (Top-5). Subsequently, two clinical studies were conducted with Autoderm with earlier models in Sweden on Caucasian skin, and in Uganda on black skin (skin type 6 on the Fitzpatrick scale)^43,44^.

While some of these points suggest that ML dermatology models can improve efficiency in primary care by reducing unnecessary referrals and speeding up diagnoses, additional studies are required to assess their practical use in clinical practice, as foreseen by the Medical Devices regulation in the European Union.

### Objectives

The main objective of the study is the prospective validation of an ML model as a diagnostic decision support tool for skin diseases through a feasibility study in a real PC clinical practice setting in a region of Catalonia, Spain.

The secondary objectives are: 1) evaluate the diagnostic accuracy and efficacy of the ML model in a clinical setting to determine the possibility of implementing it in a PC setting; 2) detect which skin lesions are missing in the study model; 3) estimate the rate of patients agreeing to participate in the study with the aim of using these data for future related research, 4) assess the PC professionals’ degree of satisfaction with the use of the artificial intelligence model.

## Methods

The study protocol is described in detail in a separate publication^45^; however, key elements are summarised below.

### Design

**Figure 1:**
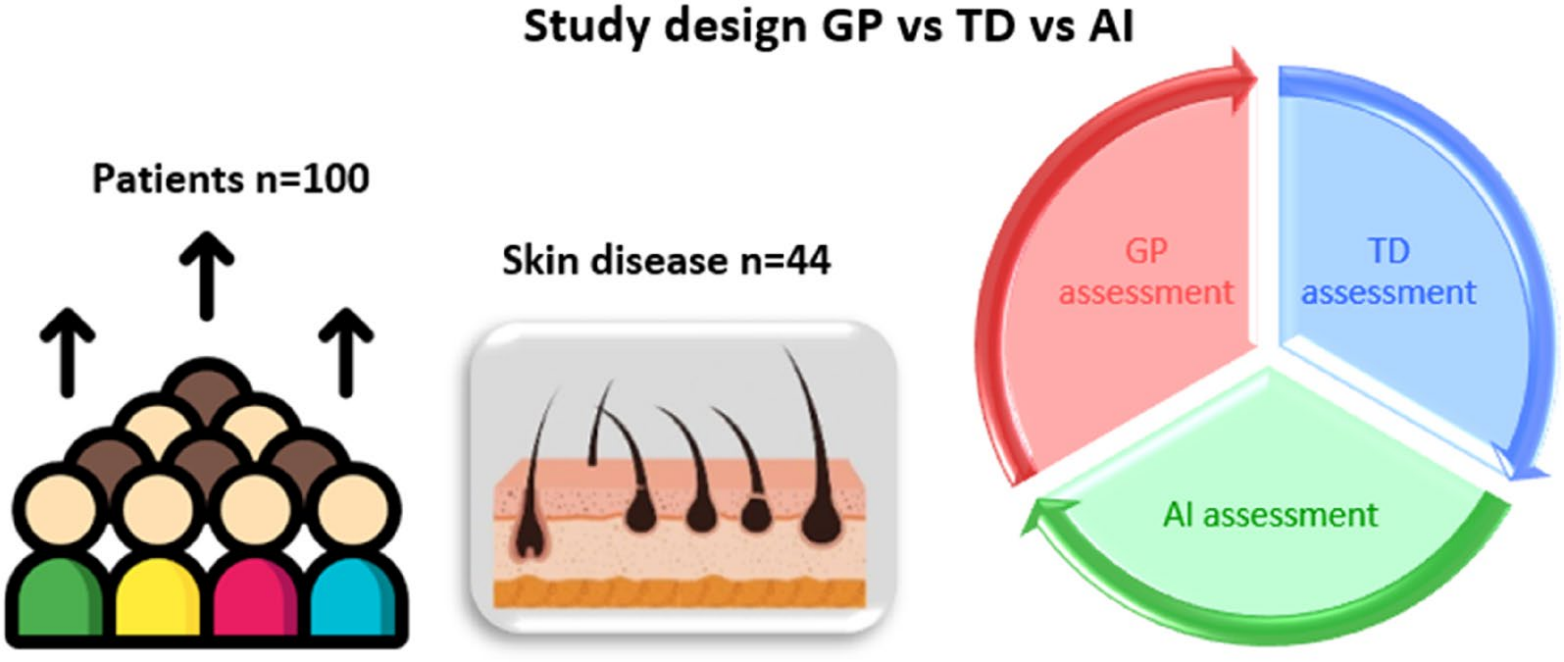
Study design general practitioner (GP) vs teledermatology (TD) vs artificial intelligence (AI).

Prospective multicentre observational feasibility study with 100 consecutive patients who consulted PC for a skin lesion in the area of Central Catalonia. Anonymised photographs of the lesions were taken and entered into the Autoderm model interface to obtain the diagnoses through AI and to be able to evaluate the diagnostic accuracy, sensitivity and specificity with respect to that of the GPs and dermatologists of the two referral hospitals (Fig. 1).

### Study population

The study was conducted in 6 PC Centres managed by the Institut Català de la Salut (main provider of PC services in Catalonia) in Central Catalonia, specifically in the regions of Bages, Berguedà and Moianès, predominantly rural and semi-rural areas. In addition, eleven GPs were invited to participate, and all accepted. The reference population included in the study was 512,050 inhabitants.

*Inclusion criteria* persons ≥ 18 years old consulting PC for a skin disease and signing the informed consent form.

*Exclusion criteria* individuals with a skin lesion that could not be photographed with a smartphone or who had difficulty understanding and complying with the protocol were excluded from the study. Poor quality images were also excluded.

### Sample size and sampling procedure

**Figure 2:**
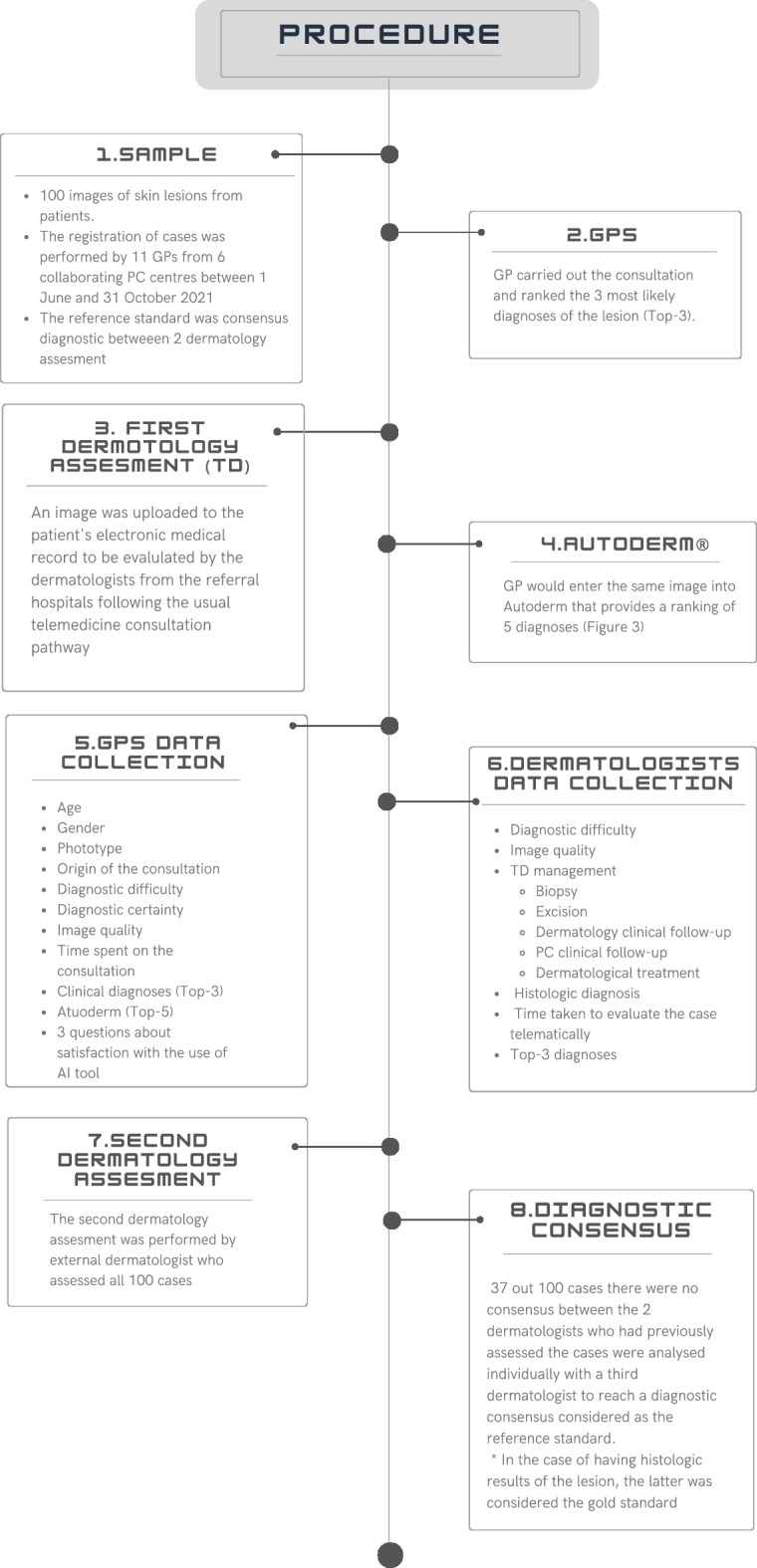
Diagram procedure. GPs: general practitioners; PC: primary care; TD: teledermatology; AI: artificial intelligence.

The sample size and sampling procedure is described in detail in a separate publication^45^; however, key elements are summarised in the Fig. 2.

As described in the study procedure (Fig. 2), the GP first made his/her diagnosis (Top-3) and then ran the image through the AI model. Likewise, in the three subjective questions on the use of the tool (Table 5), the GP was asked, whether seeing the results of the model (Top-5), had helped they with the diagnosis or differential diagnosis, or whether it had saved they the need for a teledermatology (TD) consultation.

**Figure 3:**
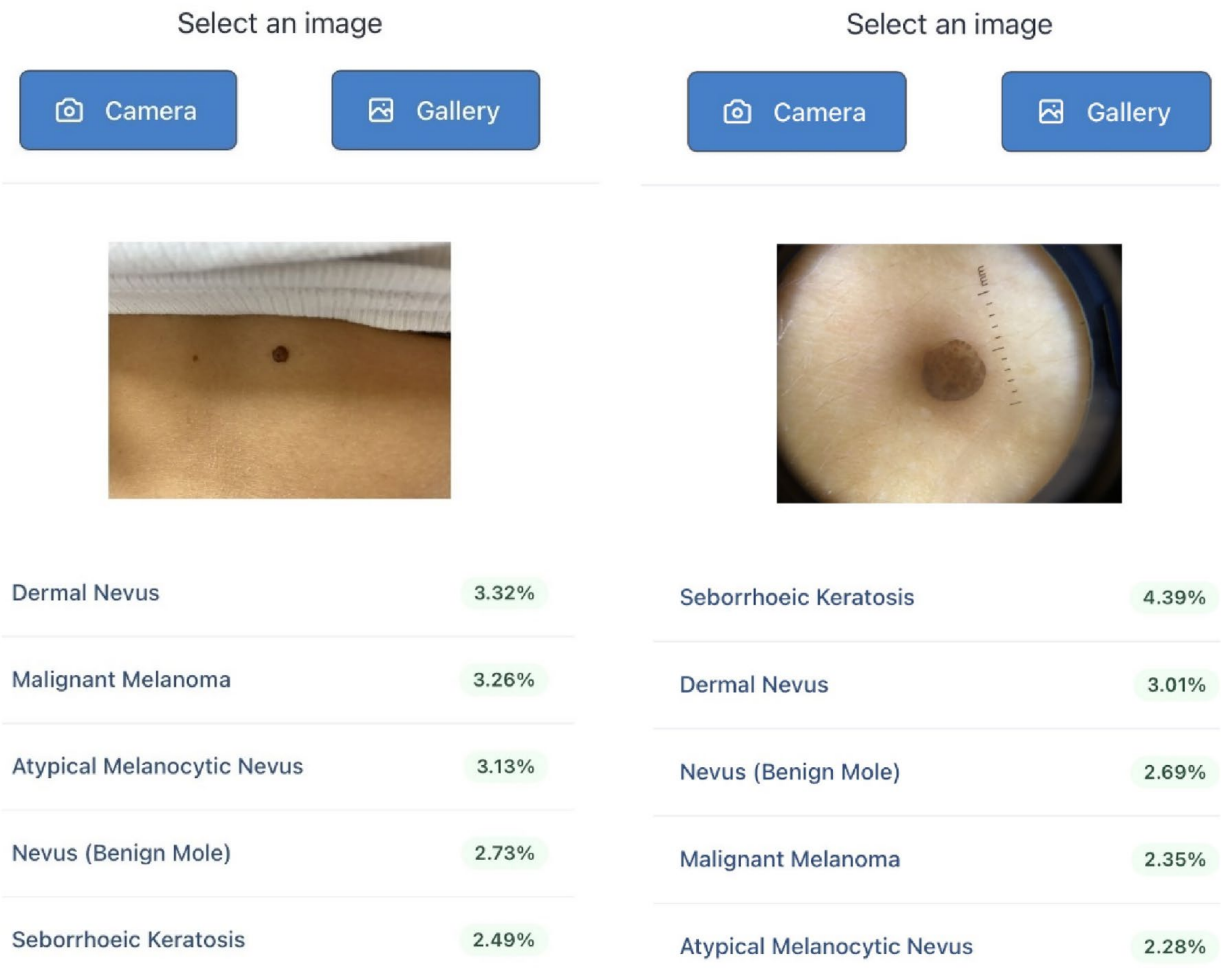
Autoderm screenshot.

Most of the photographs analysed in the study were taken by the GP during the face-to-face consultation (n = 93), as Fig. 3. The remaining 7 photos were taken by the patient and sent using the eConsultation system (The Telematic Consultation System is an asynchronous telemedicine service between patients and health professionals, integrated into the computerised information systems of the Catalan public health system)^46^. It is available to all patients and all primary care professionals.

Dermatologist 1's diagnoses are described as TD in the study.

The gold standard was defined as agreement between the top 1 diagnosis of Dermatologist 1 (Dermatologist of the reference hospital in the area, which assessed the TD according to the usual clinical model) and Dermatologist 2 (independent Dermatologist, which assessed the 100 cases only seeing the images). If both dermatologists agreed, this was considered the gold standard diagnosis for the case. Otherwise (37 cases in total), a third dermatologist reviewed the images and agreed with one of the diagnoses issued by dermatologist 1 or 2.

### Statistical analysis

The proposed sample size is based on the sample size calculation used in similar research and taking into account that it is a pilot study to validate the usefulness of the tool^44,47,48^.

The validation dataset includes 100 cases, and 4 assessments: face-to-face assessment by the GP (Top-3), assessment of the 5 differential diagnoses in order of probability from the ML model (Top-5), TD assessment by dermatologist 1 (Top-3), and assessment from the dermatologist 2 (Top-3). The evaluation of the ML model was limited to 44 types of skin diseases, while other diagnoses could be included in the evaluations of both GPs and dermatologists according to medical criteria (category other).

Regarding the five suggested diagnoses, the AI is not precise enough to only present the top three. However, with the top five diagnoses, it is estimated that the conditions are represented 95% of the time^43^. The AI serves as a search engine or analytics engine to provide differential diagnoses for skin diseases, empowering the GP to make informed decisions.

A confusion matrix was used to calculate the accuracy, sensitivity and specificity of the overall ML model and for each skin disease.

All statistical analyses were performed with R Core Team (2022). R: A language and environment for statistical computing. R Foundation for Statistical Computing, Vienna, Austria. URL https://www.R-project.org/. The confidence intervals were 95%.

### Ethical approval

Primary care GPs’ assessment and decisions were not influenced by this study, as the normal dermatology referral workflow was not affected. This project was approved by the Research Ethics Committee (REC) from the Foundation University Institute for Primary Health Care Research Jordi Gol i Gurina (IDIAPJGol) (P20/159-P) and the REC of the Hospital Sant Bernabé de Berga. A collaboration agreement has been established between the collaborating institutions: IDIAPJGol; Salut Catalunya Central, Hospital de Berga, Althaia, Xarxa Assistencial Universitària de Manresa and the company First Derm (iDoc24 Inc). The study was performed in accordance with relevant guidelines/regulations, and informed consent was obtained from all participants. All research have performed in accordance with the Declaration of Helsinki.

## Results

### Description of the sample

One hundred cases were analysed for external validation of the ML model. The PC consultations were mostly in person (93%); however, it is noteworthy that in 7% of the cases, the patient chose to send a photograph of the skin lesion and have a virtual PC consultation. The patients included in the study were mostly Fitzpatrick phototype III (n = 78) and phototype II (n = 17) (Table 1).

**Table 1:** Descriptive characteristics of the cases analysed.

	PC [n (%)]	TD [n (%)]	GS [n (%)]
*Difficulty*			
High	11 (11)	20 (20)	0 (0.0)
Average	36 (36)	40 (40)	0 (0.0)
Low	53 (53)	40 (40)	100 (100)
*Certainty*			
Yes	40 (40)	–	–
No	60 (60)	–	–
*Image quality*			
Poor	3 (3)	3 (3)	58 (58)
Average	45 (45)	9 (9)	0 (0.0)
Excellent	52 (52)	88 (88)	42 (42)
Time*	10.3 (2.74)	6.17 (2.26)	-
*Phototype*			
I	1 (1)	–	–
II	17 (17)	–	–
III	78 (78)	–	–
IV	3 (3)	–	–
V	1 (1)	–	–
*Origin*			
eConsulta	7 (7)	–	–
In person	93 (93)	–	–
*Management*			
Biopsy	–	7 (7)	–
Excision	–	7 (7)	–
Dermatology clinical follow-up	–	28 (28)	–
PC clinical follow-up	–	53 (53)	–
Dermatological treatment	–	5 (5)	–

*PC* Primary care, *TD* Teledermatology, *GS* Gold standard.

Variables described by relative frequency and percentage n (%).

*Minutes. Mean and standard deviation.

Variables that were not asked to all groups of professionals have been marked with the symbol.

Both dermatologists and GP agreed that most of the cases assessed (80% and 89%, respectively) were of low or moderate difficulty. In 88% of the cases, they considered that the quality of the image taken by the GP and evaluated by the dermatologists who resolved the telematic consultation was excellent. The photos taken by the patients it has to take into account that 4 of the 7 images were of excellent quality and 3 were of poor quality. The time needed to resolve the consultation was also evaluated, and this was higher in the case of PC (10.3 min on average) versus the time taken with TD (6.17 min on average) (Table 1). It has to take into account that the time spent on the GP consultation was estimated by each professional. It included the total time spent on a face-to-face visit. In Catalonia, a typical face-to-face visit is allotted 12 min. It is assumed that this time accounts for deductions for other tasks. However, the time spent on medical history and physical examination, as well as capturing the photo and uploading it to the shared clinical history portal for review by the referral hospital dermatologist, was included.

The total of 100 cases produced 36 different diseases or diagnoses (Table 2), of which 12 were not included in the 44 diagnoses analysed by the ML model (Online Appendix, Table 1).

**Table 2:** Description of the case studies with GS diagnosis, how many cases were studied and whether they were included in the ML model.

Diagnostics	n (%)	ML model
Acne vulgaris	2 (2)	Yes
Angiokeratoma	1 (1)	
Balanitis	1 (1)	Yes
Common wart	4 (4)	Yes
Borrelia	1 (1)	Yes
Basal cell carcinoma	4 (4)	Yes
Cutaneous squamous cell carcinoma	2 (2)	Yes
Condyloma (genital wart)	1 (1)	Yes
Chondrodermatitis nodularis helicis	1 (1)	
Lymphocytic dermatitis	1 (1)	
Unspecified dermatitis	1 (1)	Yes
Dermatofibroma	3 (3)	Yes
Dyshidrotic eczema	4 (4)	
Palmar hidradenitis	1 (1)	
Scabies	1 (1)	
Fibroma	1 (1)	
Granuloma annulare	4 (4)	
Haemangioma	3 (3)	Yes
Hidradenitis	1 (1)	
Lentigo	2 (2)	Yes
Lichen planus	1 (1)	Yes
Vascular malformation	1 (1)	
Dysplastic nevus (atypical mole)	1 (1)	Yes
Melanoma	1 (1)	Yes
Nevus (benign mole)	10 (10)	Yes
Intradermal nevus	10 (10)	Yes
Onychodystrophy	1 (1)	
Onychomycosis	1 (1)	
Post-inflammatory hyperpigmentation	1 (1)	Yes
Pityriasis versicolor	1 (1)	Yes
Pityriasis rosea	1 (1)	Yes
Psoriasis	4 (4)	Yes
Seborrheic keratosis	17 (17)	Yes
Actinic keratosis	7 (7)	Yes
Rosacea	2 (2)	Yes
Tinea corporis or dermatophytosis (ringworm)	2 (2)	Yes

The results presented in Table 2 suggest that most of the diagnoses consulted in PC were related to a benign tumour; there were 20 consultations for nevus (including the category of benign mole, dysplastic nevus and intradermal nevus), 17 cases of seborrheic keratosis, and 7 cases of actinic keratosis, among others. It should be noted that for the analysis of this study, actinic keratosis was included in the category of benign tumours, although acknowledging the potential risk of malignancy around 1%.

The second most frequent diagnostic group was inflammatory diseases with 4 cases of each of the following pathologies: psoriasis, dyshidrotic eczema and granuloma annulare and 2 cases of acne vulgaris and rosacea. This was followed by infectious diseases, with 4 cases of verruca vulgaris and 2 cases of tinea corporis. Seven cases of malignant tumours were evaluated: 1 melanoma, 4 basal cell carcinomas (BCC) and 2 cutaneous squamous cell carcinomas (cSCC).

Of the 18 cases in which the diagnosis was not included among the 44 diagnoses in the model (Online Appendix, Table 1), the diagnoses of granuloma annulare (n = 4) and dyshidrotic eczema (n = 4) are noteworthy because of the number of cases observed. Diagnoses such as scabies, fibroma, onychodystrophy, onychomycosis and hidradenitis, although only identified in 1 or 2 cases during the study, are usually seen in PC consultations and were not included in the list of diagnoses in the ML model. Of these 18 cases, 3 were histopathologically diagnosed: one haemangioma, one case of granuloma annulare and one case of lymphocytic dermatitis.

### Accuracy and sensitivity (Table 3)

**Table 3:** Overall diagnostic accuracy of artificial intelligence, teledermatology and primary care.

	Accuracy	95% CI	Sensitivity	95% CI	Specificity	95% CI
*Top 1*						
AI	0.39	(0.29; 0.49)	0.36	(0.24; 0.49)	0.98	(0.97; 0.99)
AI PCD	0.28	(0.17; 0.43)	0.34	(0.15; 0.53)	0.96	(0.94; 0.98)
TD	0.72	(0.62; 0.80)	0.7	(0.58; 0.83)	0.99	(0.98; 0.99)
PC	0.64	(0.54; 0.73)	0.61	(0.48; 0.73)	0.99	(0.98; 0.99)
*Top 3*						
AI	0.61	(0.51; 0.71)	0.52	(0.37; 0.66)	0.98	(0.96; 1.00)
AI PCD	0.61	(0.47; 0.75)	0.57	(0.34; 0.80)	0.97	(0.92; 1.00)
TD	0.90	(0.82; 0.95)	0.88	(0.80; 0.97)	0.99	(0.99; 1.00)
PC	0.76	(0.66; 0.84)	0.7	(0.57; 0.83)	0.99	(0.98; 1.00)
*Top 5*						
AI PCD	0.75	(0.61; 0.86)	0.63	(0.39; 0.87)	0.98	(0.95; 1.00)
AI	0.72	(0.62; 0.80)	0.59	(0.44; 0.75)	0.99	(0.98; 1.00)

Mean sensitivity and specificity for each of the diagnoses.

*AI* Artificial intelligence, *AI PCD* Artificial intelligence polarised light contact dermoscopy, *TD* Teledermatology, *PC* Primary care.

The diagnostic accuracy score of the ML model in Top-1 was 0.39 (0.29–0.49) compared to 0.72 (0.62–0.80) for TD and 0.64 (0.54–0.73) for GPs. These values increase significantly when Top-3 is assessed with a diagnostic accuracy of 0.61 (0.51–0.71) for the ML model and reaching 0.72 (0.62–0.80) for Top-5 (Table 3).

It should be noted that all the values of the diagnostic accuracy of the ML model are lower than those of the professionals, both for TD dermatologists and PC GPs. However, there were 18 cases in which the model was not able to recognise the disease, as it was not trained for the particular diagnosis. Thus, a subanalysis was performed including only the 82 cases corresponding to any of the 44 diagnoses with which the model was trained, after which the diagnostic accuracy increased to 0.48 (0.37–0.59) in Top-1, to 0.75 (0.66–0.85) in Top-3 and to 0.89 (0.79–0.95) in Top-5 (Table 4).

**Table 4:** Accuracy, sensitivity and specificity of the ML model with diagnoses for which it has been trained (n = 82).

	Accuracy	95% CI	Sensitivity	95% CI	Specificity	95% CI
*Top 1*						
AI	0.48	(0.37; 0.59)	0.56	(0.40; 0.72)	0.98	(0.97; 0.99)
*Top 3*						
AI	0.75	(0.66; 0.85)	0.79	(0.67; 0.91)		(0.97; 1.00)
*Top 5*						
AI	0.89	(0.79; 0.95)	0.9	(0.82; 0.98)	0.99	(0.97; 1.00)

*AI* Artificial intelligence, *TD *Teledermatology, *PC *Primary care.

The overall sensitivity of the model follows a similar trend to the diagnostic accuracy with 0.36 (0.24–0.49) in Top-1, 0.52 (0.37–0.66) in Top-3 and 0.63 (0.39–0.87) in Top-5. Compared to those of both dermatology and GP, the results are slightly lower, with 0.70 (0.58–0.83) and 0.88 (0.80–0.97) for TD Top-1 and Top-3, respectively, and 0.61 (0.48 -0.73) and 0.7 (0.57–0.83) for PC Top-1 and Top-3, respectively (Table 3).

However, it should be noted that the specificity at all levels (AI, TD and PC) is close to 1 (0.96–0.99) (Table 3).

**Figure 4:**
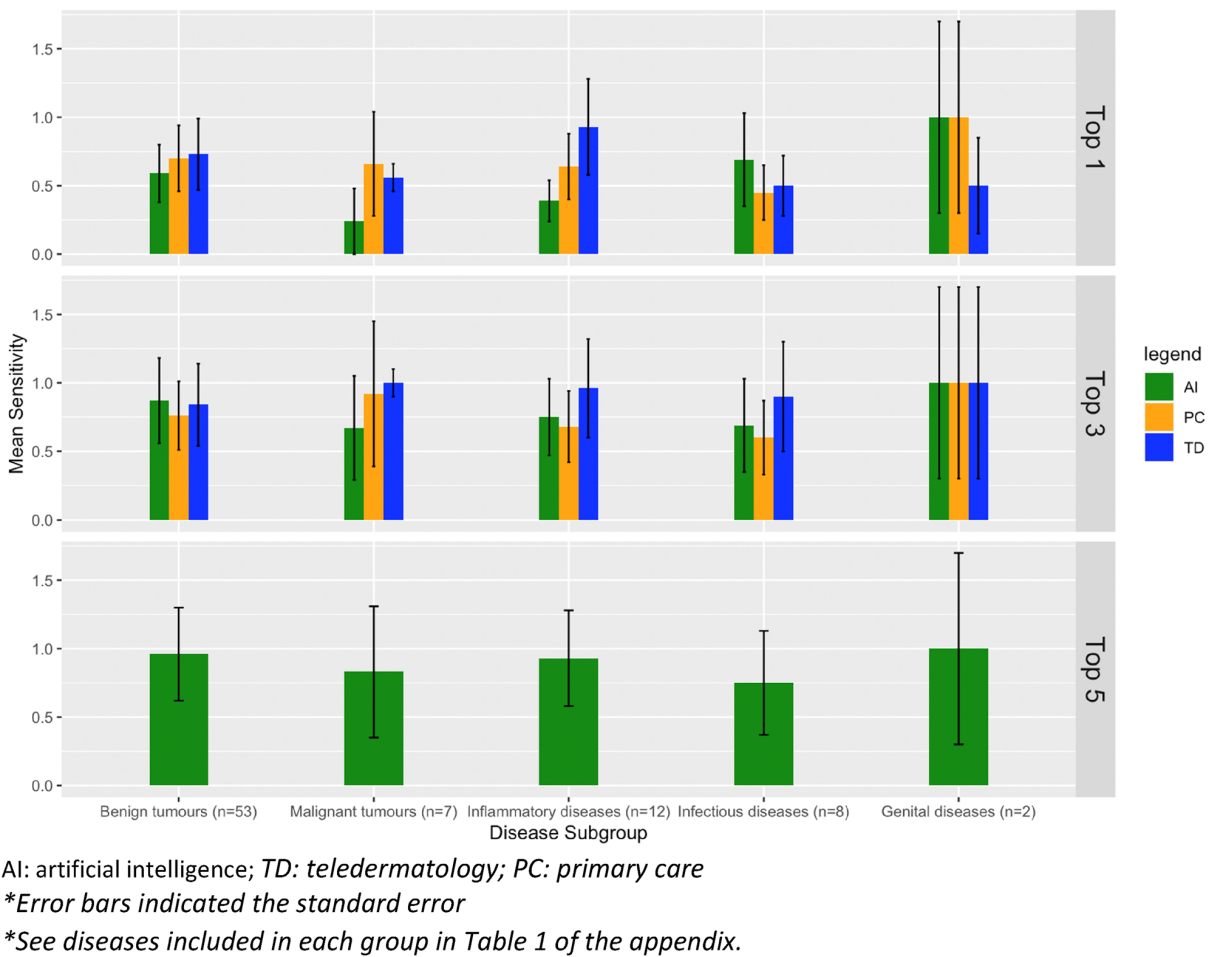
Mean sensitivity grouped by disease subgroups, only of the 82 cases recognised by the ML model.

A detailed study of sensitivity by disease was conducted (Annex, Table 2), but considering the small number of cases of some diseases, they were grouped by diagnostic groups (Fig. 4).

It was found that in the Top-3, the mean sensitivity of the model was slightly higher with respect to both PC and TD professionals in benign tumours (n = 53), where the mean sensitivity of the model was 0.87 (0.72;1.0) in the Top-3 and 0.96 (0.90; 1.0) in the Top-5, compared to 0.76 (0.63;0.89) and 0.84 (0.67;1.0) in the Top-3 for PC and TD professionals respectively.

For inflammatory diseases (n = 12), AI was only superior to GP (Top-3 0.68 (0.24;1.0)) in the Top-3, but in none of the scenarios was its accuracy superior to dermatologists (Top-3 0.96 (0.87;1.0)).

For infectious diseases (n = 8), the diagnostic accuracy of the ML model (Top-3 0.69 (0.09;1.0) and Top-5 0.75 (0, 29;1,0)) was superior to that of GP (Top-3 0.60 (0.0;1, 0)), but not compared to dermatologists (Top-3 0.90 (0.48;1,0)).

For malignant tumours, GP had a diagnostic sensitivity of 0.92 (0.56–1.0) in the Top-3, superior to that obtained by the AI, which was 0.67 (0.0;1.0) and 0.83 (0.11;1.0) in the Top-3 and Top-5, respectively. Analysing the diagnoses included in this subgroup individually, we can see that in the case of melanoma (n = 1) the sensitivity is 1 at all levels (PC, TD and AI). For cSCC (n = 2), the sensitivity in the Top-5 of the model and the Top-3 of the professionals was 1 in all cases. For BCC (n = 4), GP have a higher sensitivity in the Top-3 (0.75) compared to the model (0.5), which does not increase in the Top-5 either. In all cases, the gold standard in these 7 cases was the histopathological analysis.

For genital diseases, there was only 2 cases with an average sensitivity of 1.

During data collection, and following standard clinical practice, the 11 GP could include, if they considered it appropriate for case orientation, a dermoscopic image of the skin lesion (AI PCD), taken with a Dermlite DL100 dermatoscope or a DL200 HR applied manually to the smartphone. This situation occurred in 52% of cases, the vast majority of which corresponded to benign (39 of the 52 cases) and malignant (6 of the 7 cases) tumours.

In cases in which the GP also assessed the dermoscopic image of the lesion with the ML model, the diagnostic sensitivity of the ML model with respect to the clinical image of the same lesion increased in the following diseases: verruca vulgaris, cSCC (Top-1, Top-3 and Top-5) and intradermal nevus (Top-3 and Top-5) (Online Appendix, Table 2).

### Degree of satisfaction of the professionals

Table 5 shows the satisfaction of GPs evaluated through 3 subjective binary response questions to evaluate the satisfaction with the use of AI as a DST for each case. The 92% of GP responded affirmatively to the question of whether it helped them in the differential diagnosis approach.

**Table 5:** Satisfaction and acceptance of the GPs.

	n (%)
Together with your diagnostic criteria, would the use of AI have been sufficient to resolve the consultation without a teledermatology consultation?	
Yes	34 (34)
No	63 (63)
DK/NC	3 (3)
Did the use of AI help you with the diagnosis?	
Yes	60 (60)
No	38 (38)
DK/NC	2 (2)
Did the use of AI help you to think about other differential diagnoses?	
Yes	92 (92)
No	8 (8)

In 60% of the cases, the AI tool was helpful in reaching the diagnosis of the lesion. In the 34% of cases, they could have avoided the TD consultation (Table 5).

## Discussion

In this study, a pilot external validation test of an ML model that identifies 44 skin diseases that represent a very frequent reason for PC consultation was performed in a PC setting. This is a feasibility study in routine clinical practice and will help us to develop additional studies with a larger sample which may contribute to improve the ML model used in PC. The results have shown that the 100 cases included in the study were predominantly of phototype type III, and to a lesser extent type II. According to the new Medical Device Regulation^30^, it is imperative to perform proper evaluations of ML models for dermatology imaging applications^32^, also in all skin phototypes. Thus, more studies are needed in order to ensure that they are trained in an inclusive and balanced way, and thus perform with the same accuracy on any skin phototype to avoid the possibility of disadvantaging certain groups of people. Studies exploring the use of ML models as a diagnostic tool in the medical field are starting to be conducted, primarily in image interpretation. This includes applications in interpreting retinal imaging and chest radiography^49–51^

The overall diagnostic accuracy of the model in this study is lower than that of both GPs and the TD assessment, as well as the one obtained in the theoretical diagnosis in the proof of concept of the model^39^. However, the average diagnostic sensitivity improves substantially when analysing the 82 cases in which the gold standard is included in one of the 44 diagnoses for which the model is trained. Thus, the observed results highlight the importance of determining the diagnoses not included in order to train the model and adapt it to routine clinical practice. These results differ from most theoretical and retrospective studies in which AI accuracy is usually equal to or higher than that of clinicians^22,25,26,37^, and are consistent with the few existing prospective and real-world studies^52^. In addition, it is of relevance that the specificity of the application of AI in dermatologic imaging was very close to 1, which suggests that it is a useful tool for application in routine clinical practice as a CDST. The AI model was trained using images from an online dermatology service (First Derm), not clinical images, and the patients and images have not been verified in a clinical setting. This may result in a bias in image quality due to the technology used, even with the prevalence of some skin conditions.

Moreover, the fact that the diagnostic accuracy metrics increase with the Top-3 and Top-5 assessment is consistent with the usefulness in differential diagnosis, a fact already pointed out by Muñoz-López et al. in their study^52^. Recent algorithms tend to perform a ranked list of diagnoses. Aiding a differential diagnosis rather than a single diagnosis is particularly important in dermatology, where differential diagnosis is used for diagnostic-therapeutic decision-making. Furthermore, it can improve diagnostic accuracy when all diagnoses are taken into account, which is relevant in PC, where most of the time the most important thing is to know whether or not we are dealing with a potentially malignant lesion in order to assess the need or not for referral and/or prioritisation.

The fact that TD has been established for years in the PC environment of Central Catalonia as a screening method for in-person dermatology consultations could influence different variables, such as the high quality of the images collected, the consultation time and the degree of participation acceptance of citizens^9^. With regard to possible interferences in the quality of the images, in the case of dermoscopic images, it should be noted that the dermatoscopes used in the PC setting are not digital or adapted for smartphones, which could lower its quality and bias the image analysis both by the dermatologists and by the ML model.

The results suggest that a diagnostic aid for GPs in the resolution of dermatologic consultations would be a significant time-saver. GP can better orient the consultation at the time it occurs, not having to wait for the response time of the TD consultation (24–48 h), and, on the other hand, for dermatology specialists it would mean being able to focus their experience on cases that are difficult to manage in PC.

It is not possible to draw conclusions on the individual diagnostic sensitivity by disease and, therefore, it was represented by groups. However, the small number of cases in the pilot study allowed us to perform a more exhaustive analysis of the different diseases. Nonetheless, about 50% of the cases were encompassed within the same category of benign tumours, with the ML model having an advantage over the clinicians with a diagnostic sensitivity of 96% in the Top-5. In the analysis of the 3 cases in which the model failed to diagnose benign tumours, we can see that in 2 of the 3 cases, when analysing the dermoscopy of both nevi, the model included the diagnosis in the Top-5. Therefore, as far as the resolution of the case in routine clinical practice is concerned, it would have been correctly oriented. In the third case, the gold standard was intradermal nevus and, when analysing the Top-5 diagnosis, the ML model included the diagnosis of nevus, but not intradermal nevus, so in the overall analysis it was considered erroneous despite the fact that in clinical practice it is of no importance to differentiate between the two categories (nevus and intradermal nevus). In future versions of the ML model, these diagnoses should be considered as a single diagnosis (nevus) due to the lack of clinical relevance. Therefore, one could infer that the ML model’s diagnostic sensitivity in routine clinical practice in the Top-5 for benign tumours is 100%.

For malignant tumours, at a theoretical level the use of the ML model would not imply a diagnostic improvement. However, the results are not statiscally significant since the number of cases analysed was very small (n = 7) and the average diagnostic sensitivity of the professionals was very high in the Top-3.

In the Top-5, an average model sensitivity of 83% was observed. The ML model did not include the diagnosis of the lesion in 2 of the 7 cases of malignant tumours. These cases were one BCC and one cSCC, and the pathology report of the lesion was used as the gold standard. This case also generated diagnostic doubt among PC clinicians, since in the case of cSCC was classified as melanoma, as did the ML model. At this point, we also believe it is important to highlight that the diagnoses included in the Top-5 of the image evaluation in all cases included diagnoses in the category of malignant tumours, thus considering the malignant potential of the lesion, a relevant fact for the diagnostic and referral approach of GP.

For infectious diseases, the sensitivity of the model in the Top-5 was 75%, failing in 3 of the 9 cases included. In the detailed analysis we see that two of the cases were verruca vulgaris. One on the face, with the clinical image, the ML model diagnosed a benign tumour (nevus, intradermal nevus and seborrheic keratosis), epidermal cyst and herpes simplex, but when including the dermoscopic image, the diagnosis of verruca vulgaris was the Top-1. Therefore, showing another case that would be solved following the clinical practice of the GP who used a dermatoscope to help with the diagnostic. The second case the ML model failed probably because the image taken by the GPs showed several lesions, which may have confused both the AI and TD. The third case was a tinea corporis of the scalp with diagnostic agreement between the 3 clinicians who assessed the image; the model’s Top-5 were seborrheic dermatitis, folliculitis, neurodermatitis, vitiligo and psoriasis. Photographing the scalp is always challenging, as cameras usually focus the hair and not the scalp, where most dermatologic diseases actually reside. Therefore, it is possible that the images used for training the ML model would have incurred this problem, decreasing its diagnostic accuracy^53^.

For inflammatory diseases, the sensitivity of the Top-5 model was 93%, failing in 1 of the 11 cases. The case was acne vulgaris, in which different erythematous papular rashes could be seen, some of them with superficial crusting in the beard area. In this case, the 5 diagnoses issued by the model were: rosacea, impetigo, folliculitis, BCC and perioral dermatitis, most of them falling into the inflammatory or infectious disease category.

For genital diseases, only 2 cases were included; one of balanitis and one of condyloma, in both cases the model found the correct diagnosis in the Top-1. Despite the small number of cases included in this category, the high diagnostic sensitivity in genital diseases could be explained by the fact that the model was trained at a theoretical level with 30% of genital disease photographs in the dataset.

It is difficult to consider the optimisation of the model with the inclusion or exclusion of diagnoses to make it more accurate in routine clinical practice; however, there are diseases documented as absent, such as, for example, dyshidrotic eczema, granuloma annulare, scabies, fibroma and hidradenitis. Taking into account the authors’ clinical experience, we suggest including these diseases in future versions of the model to improve its performance.

A terminology review of the terms used by Autoderm was performed, as some of the terms used are obsolete or inaccurate in clinical practice. For example, the term "unspecified dermatitis" has never been used among dermatologists, as it is a very unspecific term. As for vascular malformations, it only takes into account haemangiomas, which would be paediatric vascular malformations, but a case assessed in adulthood was also specified. We also suggest unifying the term "Borrelia" and "erythema migrans" to avoid confusion. A proposal has also been made to improve the subclassification of acquired nevi to: junctional nevus (flat mole), compound nevus (flat mole with central raised area), intradermal nevus (raised mole) and nevus with atypical clinical features (since the diagnosis of atypia is histological).

The gold standard in this study was defined as a diagnostic consensus between two or three dermatologists, a fact that may generate, in isolated cases of high diagnostic complexity, a greater difficulty compared to studies in which the histopathological analysis of all lesions is compared. These were isolated cases that, with careful deliberation among experts, were resolved correctly, reinforcing our will to act in routine clinical practice without having to perform biopsies that would imply unnecessary morbidity.

As for the technical side of the ML model, it should be noted that one of the main advantages is that it can continue to learn patterns indefinitely as more images are obtained. This is in contrast to the normal training period for a GP. This process takes several years and some of the information and experience gained during the working life is eventually lost. A neural network can learn and work indefinitely. Everything suggests that the ML models’ constant learning could also have a positive impact on the professionals’ continued training, who would use it as a DST.

On the other hand, it is important to mention the explainability aspect. Many automatic diagnostic algorithms do not have mechanisms for communicating why a prediction is made. This leaves the observer with only a percentage probability, which is insufficient to assess whether the decision has been made correctly or not.

### Limitations

The most relevant limitation of the study is the number of images used (n = 100) for the performance evaluation of the ML model. Since Autoderm evaluates 44 skin conditions, and considering that the prevalence of a significant number of these conditions represent less than 1–5%, the sample data for each class may be unbalanced and some conditions may not be evaluated, leading to an insufficient confidence level and less conclusive results for these conditions.

Secondly, due to the size of the sample and the consecutive collecting of cases, no representative results were obtained for less frequent diseases. However, we have included most of the spectrum of skin lesions that are a common reason for PC consultation, as well as banal lesions to avoid selection bias.

Thirdly, it should be taken into account that the GPs who agreed to participate voluntarily in the study show an interest in dermatology. Not all of them have a higher academic training in the subject, but it could explain in part that the diagnostic accuracy was higher than that reported in the literature (6,7). In this context, the ML model would be at a disadvantage in the comparison of overall diagnostic accuracy and sensitivity, as well as in the analysis by disease subgroups.

Fourth, a diagnosis made with a single image may have inherent limitations compared to diagnoses made in a clinical setting. The result of the ML model was based on a single photograph, which differs from other ML models, which consider more than one photograph.

Finally, the majority of phototypes in the population where the present study was conducted are type II and III, which could be related to a decrease in diagnostic accuracy, as the other two clinical studies with Autoderm were conducted in Sweden (type I and II) and Uganda (type VI) (44,45).

Finally, although it is a strength of the study to know that all GPs accepted to participate in the study, it must be taken into account that it is not possible to know the number of patients invited to participate in the study because the GPs did not register the patients who did not accept to participate in the study.

## Conclusions

This external validation feasibility study provides significant advances with respect to previous studies regarding the application of AI in routine clinical practice in PC. It provides, in first place, the diagnostic accuracy results of the ML model for images taken by different GPs in real conditions, including benign or malignant tumours and inflammatory, infectious and genital diseases. In addition, the degree of satisfaction of the professionals with the use of the AI tool in the diagnosis and also with the usefulness of having the differential diagnosis were also recorded.

Despite the fact that the diagnostic accuracy in real conditions was lower than the theoretical accuracy of the ML model itself and of the professionals in most diagnostic categories, the results highlight the need for more prospective studies in clinical practice for external validation of the ML models and to be able to assess their implication in improving clinical practice in a real environment. It is necessary for technicians and clinicians to work together to improve the software and adapt it to the clinical environment. A paradigm shift is needed in the theoretical evaluation metrics of these ML model to include clinical and satisfaction parameters adapted to the real world, as called for in the new European Medical Devices Regulation.

Because of its accessibility and proximity to the public, as well as the diagnostic diversity of the diseases, PC is an area to be taken into account in future AI studies. AI as a DST can provide greater diagnostic accuracy for GPs, saving time and money by reducing waiting lists for dermatology and optimising the time that dermatology specialists can devote to the most complex cases, maintaining the quality, safety and satisfaction of professionals and citizens in the resolution of consultations related to skin lesions.

## Supplementary Information

Supplementary Information.

## Data Availability

The datasets generated and/or analysed during the current study are not publicly available because our manuscript was based on confidential and sensitive health data but are available from the corresponding author on reasonable request.
