# Supplementary material for: Exploring the potential of artificial intelligence in improving skin lesion diagnosis in primary care
Source: Sci Rep. 2023 Mar 15;13:4293. doi: 10.1038/s41598-023-31340-1 (PMC10015524; doi:10.1038/s41598-023-31340-1)
Supplement: Supplementary file 1 — Supplementary Information. [file 41598_2023_31340_MOESM1_ESM.docx]

**Table 1. List of diagnoses included in the Machine learning model.**

| **Benign tumours** |
| --- |
| Dermatofibroma |
| Haemangioma |
| Lentigo |
| Dysplastic nevus (atypical mole) |
| Nevus (benign mole) |
| Intradermal Nevus (Dermal Nevus) |
| Seborrheic keratosis |
| Actinic keratosis |
| Epidermal/benign cyst |
| Fordyce spots or granules |
| **Malignant tumours** |
| Basal cell carcinoma |
| Malignant melanoma |
| Cutaneous squamous cell carcinoma |
| **Inflammatory diseases** |
| Acne vulgaris |
| Atopic dermatitis |
| Contact dermatitis |
| Unspecified dermatitis |
| Perioral dermatitis |
| Seborrheic dermatitis |
| Post-inflammatory hyperpigmentation |
| Lichen planus |
| Lichen simplex chronicus |
| Chilblains (pernio) |
| Insect bite |
| Pityriasis rosea |
| Psoriasis |
| Rosacea |
| Urticaria |
| Vitiligo |
| **Infectious diseases** |
| Verruca vulgaris (common wart) |
| Borrelia |
| Folliculitis |
| Furuncle (deep folliculitis) |
| Herpes simplex |
| Herpes zoster |
| Impetigo |
| Molluscum contagiosum |
| Pityriasis versicolor |
| Tinea corporis or dermatophytosis (ringworm) |
| **Genital diseases** |
| Balanitis |
| Condyloma (genital wart) |
| Genital herpes |
| Pearly penile papules |
| Syphilis |

| Table 2. Sensitivity by disease (only of the 82 AI cases) | | | | | | | | | | | |
| --- | --- | --- | --- | --- | --- | --- | --- | --- | --- | --- | --- |
|  | **PC Top 1** | **PC Top 3** | **TD Top 1** | **TD Top 3** | **AI Top 1** | **AI Top 3** | **AI Top 5** | **N PCD** | **AI PCD Top 1** | **AI PCD Top 3** | **AI PCD Top 5** |
| Actinic keratosis (7) | 0.71 | 0.71 | 0.86 | 0.86 | 0.71 | 0.86 | 1.00 | 6 | 0.17 | 0.67 | 0.83 |
| Pityriasis rosea (1) | 1.00 | 1.00 | 1.00 | 1.00 | 1.00 | 1.00 | 1.00 |  | - | - | - |
| Verruca vulgaris (common wart) (4) | 0.75 | 1.00 | 0.50 | 1.00 | 0.25 | 0.25 | 0.50 | 3 | 0.67 | 1.00 | 1.00 |
| Seborrheic keratosis (17) | 0.88 | 0.94 | 0.82 | 1.00 | 0.65 | 0.94 | 1.00 | 15 | 0.40 | 0.63 | 0.73 |
| Psoriasis (4) | 0.50 | 0.75 | 0.50 | 0.75 | 0.25 | 0.75 | 1.00 |  | - | - | - |
| Rosacea (2) | 1.00 | 1.00 | 1.00 | 1.00 | 0.00 | 1.00 | 1.00 |  | - | - | - |
| Nevus (benign mole) (10) | 0.50 | 0.80 | 0.90 | 1.00 | 0.20 | 0.70 | 0.90 | 7 | 0.14 | 0.71 | 0.86 |
| Intradermal Nevus (dermal nevus) (10) | 0.70 | 0.80 | 0.60 | 0.70 | 0.20 | 0.50 | 0.80 | 9 | 0.11 | 0.55 | 0.89 |
| Cutaneous squamous cell carcinoma (2) | 0.50 | 1.00 | 0.50 | 1.00 | 0.00 | 0.50 | 1.00 | 1 | 1.00 | 1.00 | 1.00 |
| Dyshidrotic eczema (4) | 0.25 | 0.25 | 1.00 | 1.00 | - | - | - |  | - | - | - |
| Balanitis (1) | 1.00 | 1.00 | 1.00 | 1.00 | 1.00 | 1.00 | 1.00 |  | - | - | - |
| Lentigo (2) | 0.50 | 0.50 | 0.00 | 0.50 | 1.00 | 1.00 | 1.00 |  | - | - | - |
| Angiokeratoma (1) | 1.00 | 1.00 | 1.00 | 1.00 | - | - | - |  | - | - | - |
| Condyloma (genital wart) (1) | 1.00 | 1.00 | 0.00 | 1.00 | 1.00 | 1.00 | 1.00 | 1 | 0.00 | 0.00 | 0.00 |
| Pityriasis versicolor (1) | 1.00 | 1.00 | 1.00 | 1.00 | 1.00 | 1.00 | 1.00 |  | - | - | - |
| Haemangioma (3) | 0.67 | 0.67 | 0.67 | 0.67 | 1.00 | 1.00 | 1.00 | 1 | 0.00 | 1.00 | 1.00 |
| Dysplastic nevus (atypical mole) (1) | 1.00 | 1.00 | 1.00 | 1.00 | 0.00 | 1.00 | 1.00 |  | - | - | - |
| Unspecified dermatitis (1) | 0.00 | 0.00 | 1.00 | 1.00 | 0.00 | 0.00 | 1.00 |  | - | - | - |
| Tinea corporis or dermatophytosis (ringworm) (2) | 0.50 | 1.00 | 0.00 | 0.50 | 0.50 | 0.50 | 0.50 |  | - | - | - |
| Granuloma annulare (4) | 0.25 | 0.50 | 0.75 | 1.00 | - | - | - |  | - | - | - |
| Dermatofibroma (3) | 0.67 | 0.67 | 1.00 | 1.00 | 1.00 | 1.00 | 1.00 | 1 | 1.00 | 1.00 | 1.00 |
| Basal cell carcinoma (4) | 0.50 | 0.75 | 0.25 | 1.00 | 0.25 | 0.50 | 0.50 | 4 | 0.25 | 0.50 | 0.50 |
| Malignant melanoma (1) | 1.00 | 1.00 | 1.00 | 1.00 | 1.00 | 1.00 | 1.00 | 1 | 0.00 | 1.00 | 1.00 |
| Hidradenitis (1) | 1.00 | 1.00 | 1.00 | 1.00 | - | - | - |  | - | - | - |
| Acne vulgaris (2) | 1.00 | 1.00 | 1.00 | 1.00 | 0.50 | 0.50 | 0.50 |  | - | - | - |
| Scabies (1) | 1.00 | 1.00 | 1.00 | 1.00 | - | - | - |  | - | - | - |
| Lichen planus (1) | 1.00 | 1.00 | 1.00 | 1.00 | 1.00 | 1.00 | 1.00 |  | - | - | - |
| Chondrodermatitis nodularis helicis (1) | 1.00 | 1.00 | 1.00 | 1.00 | - | - | - |  | - | - | - |
| Vascular malformation (1) | 0.00 | 0.00 | 1.00 | 1.00 | - | - | - |  | - | - | - |
| Post-inflammatory hyperpigmentation (1) | 0.00 | 0.00 | 1.00 | 1.00 | 0.00 | 1.00 | 1.00 |  | - | - | - |
| Onychomycosis (1) | 1.00 | 1.00 | 0.00 | 1.00 | - | - | - |  | - | - | - |
| Onychodystrophy (1) | 0.00 | 0.00 | 0.00 | 0.00 | - | - | - |  | - | - | - |
| Borrelia (1) | 0.00 | 0.00 | 1.00 | 1.00 | 1.00 | 1.00 | 1.00 |  | - | - | - |
| Lymphocytic dermatitis (1) | 0.00 | 0.00 | 0.00 | 0.00 | - | - | - |  | - | - | - |
| Palmar hidradenitis (1) | 0.00 | 0.00 | 0.00 | 1.00 | - | - | - |  | - | - | - |
| Fibroma (1) | 0.00 | 1.00 | 1.00 | 1.00 | - | - | - |  | - | - | - |

PC: primary care; TD: teledermatology; AI: artificial intelligence; PCD: polarised light contact dermoscopy

* (n) gold standard

Variables that were not analysed by the AI (diagnosis not included in the model) were marked with the - symbol
